# The Effect of the Frost upon Public Health

**Published:** 1861-03

**Authors:** 


THE EFFECT OF THE FROST UPON PUBLIC HEALTH.—We extract the following from the *London Observer*:—

"The late severe weather has fearfully affected the public health. The deaths in the metropolis during the last fortnight have been more than 700 above the average for the past ten years. The old especially have suffered. Many aged people, who might, humanly speaking, have survived for some years, have been cut off with terrible suddenness. The returns of the Registrar-General, when they come to be analyzed, will show a greater number of sudden deaths than have, perhaps, ever been recorded before in this country. The intense cold, in fact, paralyzed the whole system of persons stricken in years. The prevailing causes of death have been pneumonia, congestion of the lungs, and apoplexy. In the City of London the mortality has been excessive. The average number of deaths in the corresponding weeks of previous years have been 67; during the past week the deaths numbered 95, being only 7 less than the deaths in the week when the cholera was most fatal in the City of London, in 1848. Last Thursday week, when the whole of the metropolis was enveloped in a dense fog, large numbers of persons were struck down as if shot. Dr. Letheby, in his report to the City Commissioners of Sewers, says: 'the quantity of organic vapor, sulphate of ammonia, and finely-divided soot in the atmosphere was unprecedented. It amounted to nearly four grains in the cubic foot of air, and its effect on the eyes and the delicate bronchial membranes was most irritating. This is evidenced by the enormous amount of illness and mortality from acute pulmonary affections. Pneumonia, for example, has risen in the course of the last few weeks from only 1 death in the week to 11, the successive numbers being 1, 2, 5, 11, and bronchitis from 5 to 25 ' In the City of London, nevertheless, the cold has not been anything like so severe as in many other parts of the country, where the thermometer has fallen several degrees below zero; and it therefore follows, that if a less degree of cold has produced such a fearful increase of mortality, a still greater mortality may be expected where the cold was more intense. An instance of the effect of cold may be mentioned. A gentleman a few days ago was met in the street by a friend, who was so much struck by his appearanee that he asked him if he was ill. 'No,' was the reply; 'I am quite well, only I should like a little more breath.' His friend insisted upon his going home, and put him in a cab. Arrived at home, he had no sooner laid down than he became delirious, and within twenty-four hours he was a corpse. The cold had struck on his lungs, and vital congestion had followed. The average anticipatory number of deaths for the week ending the twelfth instant, in the entire metropolis, corrected according to increase of population, was 1365. The actual number was 1707, showing an excess of 342; an increase almost unparalleled, except in the case of such an epidemic as cholera. But it is not merely the cold that kills, although it is the primary cause. During the long frost, thousands and tens of thousands have been thrown out of employment, and at the very moment when they require nourishing food and warm clothing they have far less of both than on ordinary occasions. The human frame becomes consequently debilitated, and rendered more liable to the effects of the cold. The poor cannot remain at home in their wretched dwellings; they wander about listlessly, and when the cold seizes them they have not sufficient stamina to resist it."

